# Using Synbiotics as a Therapy to Protect Mental Health in Alzheimer's Disease

**DOI:** 10.4014/jmb.2403.03021

**Published:** 2024-06-21

**Authors:** Anh Pham Thi Ngoc, Adil Zahoor, Dong Gyun Kim, Seung Hwan Yang

**Affiliations:** 1 Department of Biotechnology, Chonnam National University, Yeosu 59626, Republic of Korea

**Keywords:** Alzheimer’s disease, synbiotics, anxiety, gut-brain axis

## Abstract

Alzheimer’s disease (AD) is a progressive neurological disorder that represents a major cause of dementia worldwide. Its pathogenesis involves multiple pathways, including the amyloid cascade, tau protein, oxidative stress, and metal ion dysregulation. Recent studies have suggested a critical link between changes in gut microbial diversity and the disruption of the gut–brain axis in AD. Previous studies primarily explored the potential benefits of probiotics and prebiotics in managing AD. However, studies have yet to fully describe a novel promising approach involving the use of synbiotics, which include a combination of active probiotics and new-generation prebiotics. Synbiotics show potential for mitigating the onset and progression of AD, thereby offering a holistic approach to address the multifaceted nature of AD. This review article primarily aims to gain further insights into the mechanisms of AD, specifically the intricate interaction between gut bacteria and the brain via the gut–brain axis. By understanding this relationship, we can identify potential targets for intervention and therapeutic strategies to combat AD effectively. This review also discusses substantial evidence supporting the role of synbiotics as a promising AD treatment that surpasses traditional probiotic or prebiotic interventions. We find that synbiotics may be used not only to address cognitive decline but also to reduce AD-related psychological burden, thus enhancing the overall quality of life of patients with AD.

## Introduction

Alzheimer’s disease (AD) is a progressive neurodegenerative illness that impairs neurocognitive function and impedes mental capacity development [1]. In 2020, approximately 5.8 million individuals were diagnosed with AD in America. This disease initially manifests as mild memory loss and gradually progresses to the loss of conversational ability and responsiveness to the environment. It primarily affects the parts of the brain responsible for thought, memory, and language [2].

Understanding the pathophysiology of AD and developing effective therapeutics are multifaceted fields of study. The etiology of AD involves various factors, including mitochondrial dysfunction, oxidative stress (OS), lipid peroxidation, reactive oxygen species (ROS), and DNA/RNA/protein oxidation. The progression of AD is also influenced by disruptions in energy metabolism and calcium homeostasis, as well as neuroinflammation and neuronal death [3]. Verbal and visual memory deficiencies have been associated with psychiatric illnesses such as anxiety, which are also related to decreased hippocampal volume and function in AD [4, 5]. Anxiety is an early symptom of AD, and several studies have established the correlation between anxiety and AD pathology biomarkers [6]. Individuals experiencing anxiety have shown positive amyloid scans and mesial temporal entorhinal alterations, which include atrophy, hypometabolism, and presence of neurofibrillary tangles [7]. In the context of AD, anxiety is attributed to multifactorial causes, such as neurobiological factors and psychological influences, including cognitive deficits and external stresses [6].

The concept of a “synbiotic” was initially introduced by Gibson and Roberfroid [8] to denote a synergistic combination of probiotics and prebiotics. Synbiotics were developed to overcome potential challenges in the survival of probiotics in the intestinal system and achieve the beneficial qualities of probiotics and prebiotics. Consequently, a well-balanced combination of these elements in one product yields outcomes superior to the individual activities of probiotics or prebiotics alone [2]. In recent years, studies on neurodegenerative pathology have focused on prebiotic, probiotic, and synbiotic nutrition [9]. The concept of “psychobiotics” has also emerged, thereby highlighting the mental health benefits of probiotics. The consumption of psychobiotics positively affects the gut–brain-mediated nervous system through various mechanisms, including neuroendocrine, neuroimmunological, and enteric pathways [10]. These effects help improve the functioning of the gastrointestinal and central nervous systems [10]. Numerous preclinical studies and clinical trials on AD have demonstrated the beneficial effects of probiotics and prebiotics. However, few studies have explored the use of synbiotics for the treatment of AD and mental health.

This review aims to explore the potential pathways that could serve as targets for the treatment of AD and to elucidate the health-promoting benefits of synbiotics on mental health. It also addresses the limitations that should be further studied.

## Hypotheses Describing the Fundamental Mechanisms Underlying AD

**Fig. 1:**
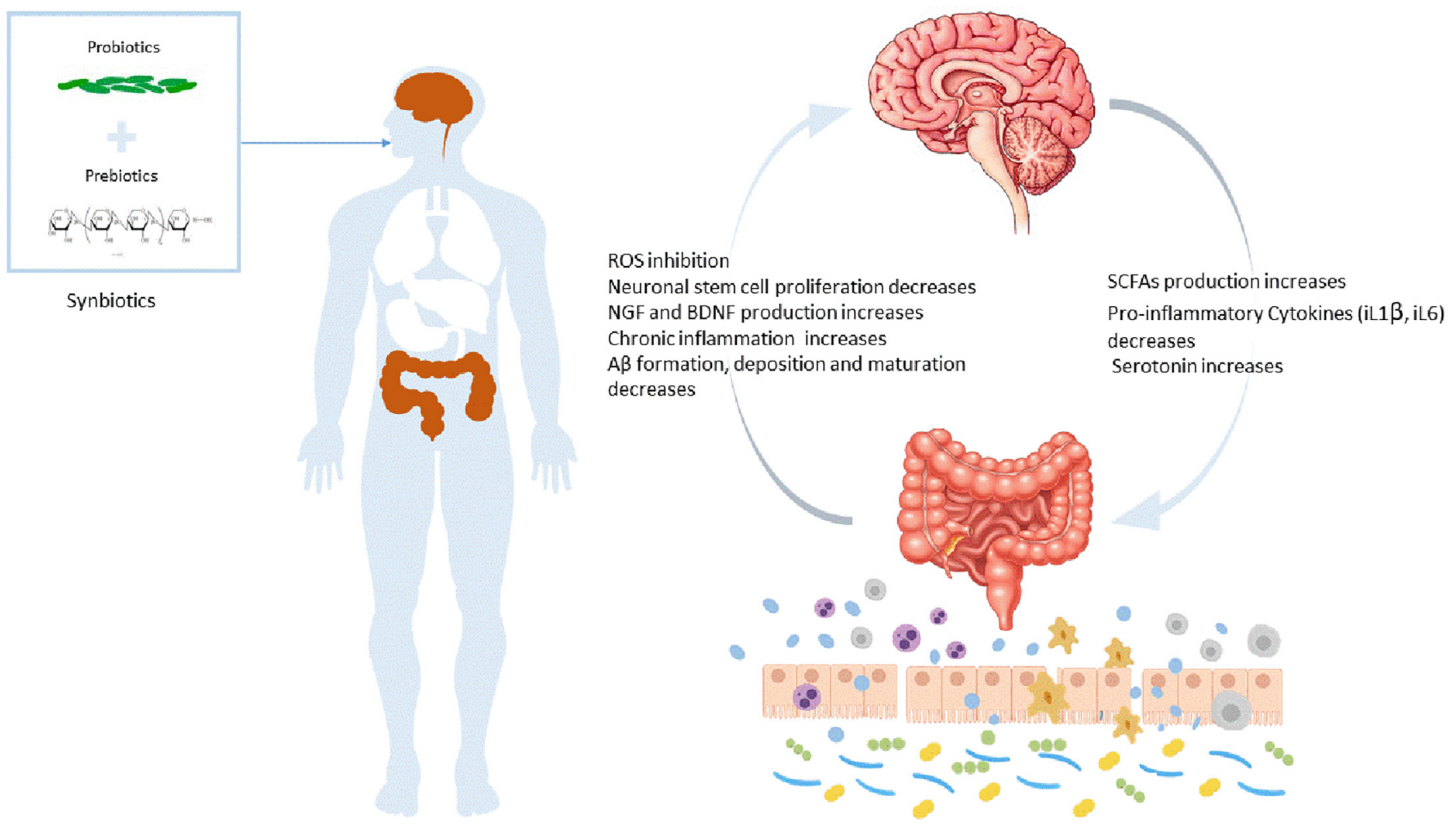
Mechanisms underlying Alzheimer’s disease. It describes how synbiotics affects Alzheimer's disease through microbiota-gut-brain axis. The good probiotics could reduce inflammation, inhibit ROS, reduce the Aβ plaque formation and increase protective molecules such as brain-derived neurotrophic factor (BDNF) and nerve growth factor (NGF). Meanwhile, signals from the brain help stimulate the production of short-chain fatty acids (SCFAs), serotonin and reduce proinflammatory cytokines in the gut.

With the understanding of the molecular causes of AD, potential medications that can inhibit the progression of this debilitating condition have been developed. Several hypotheses, including the amyloid cascade, hyperphosphorylated tau protein, metal ion, cholinergic and OS hypotheses, have been proposed as fundamental mechanisms of AD (Fig. 1). In this section, we discuss these widely accepted concepts and explore clinical studies to evaluate their validity.

## Amyloid Cascade Hypothesis

**Fig. 2:**
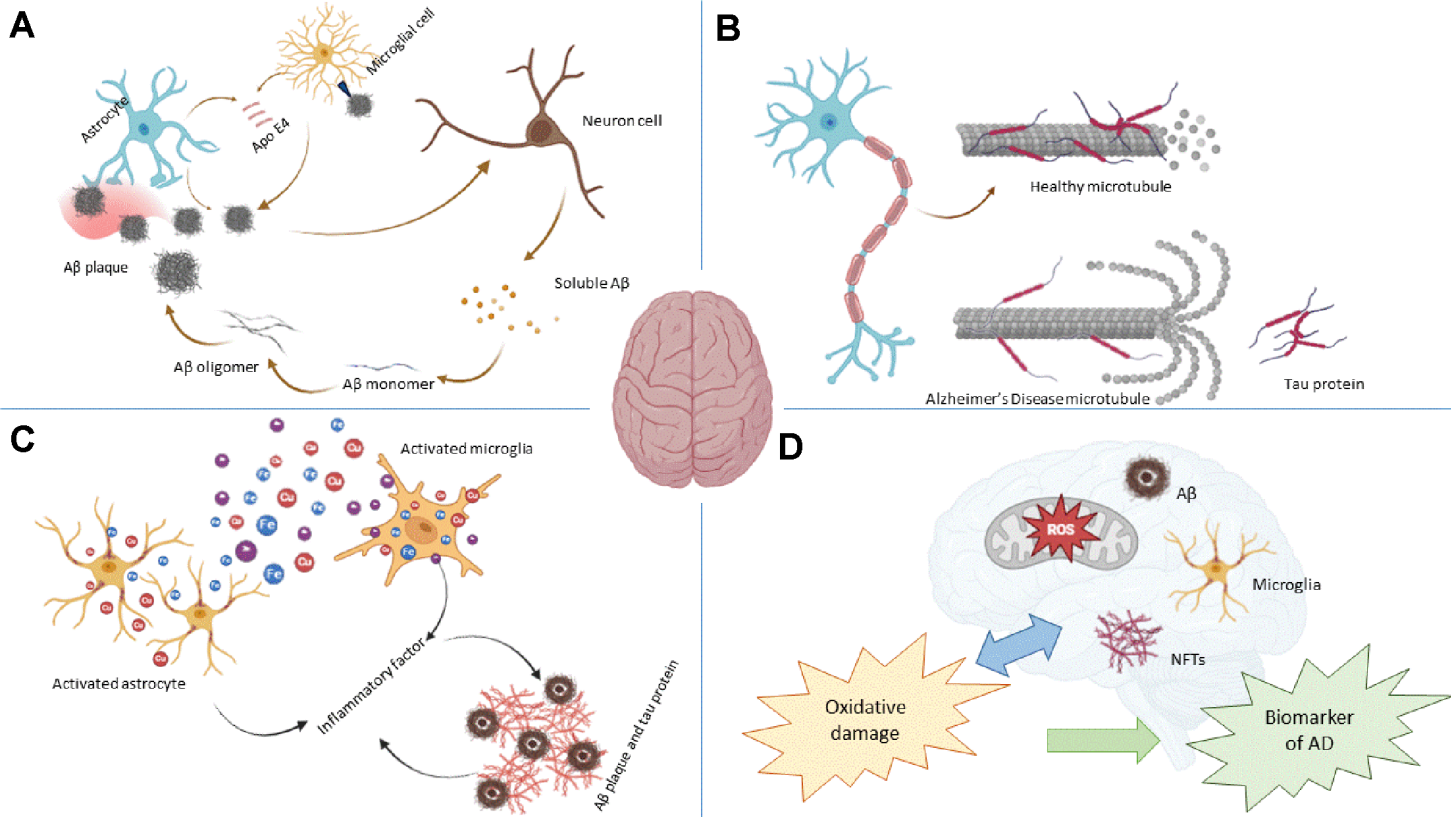
Pathogenesis of Alzheimer’s disease. (**A**) Amyloid beta (Aβ) cascade hypothesis; (**B**) hyperphosphorylated tau protein hypothesis; (**C**) metal ion hypothesis; and (**D**) oxidative stress hypothesis.

According to the amyloid cascade hypothesis, first proposed in 1991, the abnormal accumulation of amyloid β (Aβ) plaques in different brain regions contributes to neurodegeneration in AD. Aβ plaques act as triggers for a cascade of events, including neurotic damage [11], formation of neurofibrillary tangles through tau protein, neuronal dysfunction, and cell death [12]. This process stimulates microglia and astrocytes, resulting in inflammatory and oxidative responses. Ultimately, neuronal malfunction and apoptosis may lead to AD development (Fig. 2A).

Aβ can activate tau protein kinase 1; consequently, it triggers the abnormal phosphorylation of tau protein and causes the formation of paired helical filaments (PHFs) and neurofibrillary tangles (NFTs). Because of these pathological changes, the progression of tau pathology in AD further accelerates [13]. Furthermore, certain genetic factors considerably influence the onset and progression of AD [14]. Genes such as APP, PSEN-1, and PSEN-2 are strongly associated with AD pathology and can cause abnormal Aβ production [15]. Clinical research has devoted remarkable efforts to developing novel anti-AD drugs. Although approved medications provide symptomatic relief, they do not alter the disease course. These medications are often related to critical adverse effects and should only be used in severe cases. Therefore, ongoing research focuses on amyloid-based therapeutics to provide potentially effective treatments for this disease. Interventions based on the Aβ hypothesis aim to reduce Aβ production and aggregation while enhancing its clearance.

## Hyperphosphorylated tau Protein Hypothesis

Tau protein is a family of six highly soluble protein isoforms encoded by the microtubule-associated protein tau gene. They preserve the stability of microtubules in axons and are widely distributed in neurons in the central nervous system (CNS). Conversely, they are less prevalent in oligodendrocytes and astrocytes, which are immune cells in the brain [16]. When tau undergoes atypical phosphorylation, normal adult tau is converted into PHF-tau. Then, these insoluble filaments accumulate and form NFTs, a characteristic hallmark of AD [17].

Various approaches have been explored to inhibit tau protein and consequently prevent neurodegenerative diseases, including AD. In one strategy, protein kinases are modified to prevent the abnormal hyperphosphorylation of tau. By targeting these kinases, researchers aim to maintain the normal phosphorylation state of tau and prevent neurofibrillary tangle formation. In another approach, anti-inflammatory treatments are used to alleviate brain inflammation, which has been implicated in tau hyperphosphorylation and aggregation (Fig. 2B) [18].

## Metal ion Hypothesis

Metal ions, such as zinc and copper, participate in neuronal activity and the functioning of metalloproteins within synapses (Fig. 2C). These ions maintain normal brain cell function and synaptic transmission. However, disturbances in the homeostasis of metal ions or the accumulation of metals without known biological functions can disrupt the ionic balance and favor the development of neurodegenerative disorders, including AD [19].

The function of metal ions in AD has been elucidated for their use in potential therapeutic strategies. For example, the pathological effects associated with Aβ and tau protein aggregation may be mitigated by modulating metal ion concentrations or developing agents that can target metal ion imbalances. AD progression may be slowed down, and its debilitating effects may be alleviated by maintaining proper metal ion homeostasis and preventing metal-induced toxicity. However, further research and clinical investigations are needed to understand the complex relationship of metal ions with AD and to develop effective treatments targeting these mechanisms.

## OS Hypothesis

OS is characterized by the accumulation of various compounds and free radicals derived from molecular oxygen. With aging, OS increases in the brain and causes a redox imbalance, which is attributed to the overproduction of ROS or a deficiency in the antioxidant system [20]. In the context of AD, OS is implicated in disease pathogenesis, triggering free radical production through processes such as dysfunction of the mitochondria and disruption of transition metal homeostasis (Fig. 2D). These free radicals lead to neurotoxicity mediated by Aβ and tau proteins [21].

OS has been implicated as a potential source of Aβ accumulation. In neuronal and non-neuronal cells, oxidants and oxidative byproducts can enhance the expression of amyloid precursor protein (APP) and increase the intracellular and released levels of Aβ [22]. Furthermore, Aβ promotes the release of endothelin-1 (ET-1), a peptide that binds to ETA receptors and reduces ROS levels. However, this binding leads to the release of calcium ions (Ca^2+^), thereby constricting pericytes and brain capillaries. Consequently, this constriction decreases the supply of glucose and oxygen in brain tissues and potentially contributes to the development of neuropsychiatric disorders [23]. Furthermore, the homeostasis of metals, including copper, zinc, and iron, can be disrupted by OS, and such disruption is closely linked to neural activity. These disturbances in metal homeostasis and abnormal interactions between Aβ and metal ions have been implicated in the development of Aβ deposition in the brains of individuals with AD. OS also considerably affect tau protein by promoting its hyperphosphorylation and polymerization [24]. Collectively, these findings suggest that reducing or eliminating ROS can potentially delay the onset or slow the progression of AD through various mechanisms. Natural antioxidants may help prevent or treat AD by targeting multiple distinct molecular mechanisms involved in the development and progression of AD.

## Modulation of the Gut Microbiota–Brain Axis

The gut–brain axis (GBA) functions as a bidirectional communication system that connects the central and enteric neural systems and bridges the emotional and cognitive regions of the brain with peripheral functions of the intestine [25]. This axis preserves homeostasis in the digestive system, central nervous system, and gut microbiota [26]. Communication between the digestive system and the central brain involves various connections, including chemical mediators, neuronal networks, and the immune system [27].

The chemical communication between the gut microbiota and the nervous system is a critical aspect achieved through direct interactions involving short-chain fatty acids (SCFAs) and indirect chemical signaling pathways [27]. Numerous studies have emphasized that SCFAs influence the central nervous system by modulating neurotransmitter and neurotrophic factor levels. For instance, Schroeder *et al*. [28] revealed that sodium butyrate injected in mice can alter brain histone activity, thus eliciting beneficial behavioral effects. Furthermore, acetate, another SCFA, participates in appetite regulation by affecting neurotransmitters such as glutamate, glutamine, and GABA in the hypothalamus; consequently, the signal for satiety is enhanced [29].

Studies on the equilibrium of innate immune system cells within the brain have shown that the bidirectional communication between the central nervous system and gut bacteria occurs through the immune pathway [30, 31]. Certain immune cells, such as T and B cells, considerably influence brain function. For instance, T cells are associated with various complex brain functions, including spatial working memory, cognition, emotion, and stress response [32], and B cells recruit peripheral immune cells to the injured brain [33]. The blood–brain barrier (BBB) limits the access of circulating T and B cells to the underlying parenchyma; as a result, immune modulation within the CNS is mediated by microglia, astrocytes, and oligodendrocytes [34]. The gut microbiome regulates and modifies the development and functioning of microglia [35]. Therefore, understanding the effect of the gut microbiota on the immune system and the CNS can provide valuable insights into potential therapeutic interventions for neurodegenerative diseases, including AD.

## Synbiotics in Pharmaceutical Supplements for AD

Probiotics, which include bacteria that considerably affect human health, and prebiotics, which are fiber-based compounds that serve as nourishment for probiotics, can be combined to form synbiotics [36]. Well-known probiotic strains in the genus *Bifidobacteria* and *Lactobacillus* not only promote a healthy immune function and intestinal homeostasis but also inhibit neuroinflammation and enhance cognitive performance [37]. For instance, certain probiotic strains, *Bifidobacterium breve* A1, downregulate immune response genes and reduce inflammatory markers in the hippocampus of AD mouse models [38]. Moreover, they potentially improve cognitive function in elderly adults with memory issues [39].

In addition to the influence of probiotics, the effects of prebiotic components, such as fructooligosaccharides (FOS), which naturally occur in various fruits and vegetables, have been investigated in relation to cognitive deficits [40]. Chen *et al*. [40] demonstrated that FOS derived from *Morinda officinalis* not only regulates the synthesis and release of neurotransmitters in mice but also exerts preventive effects against OS and inflammatory disorders.

Xylooligosaccharides (XOS) have also been widely explored and have shown equally promising results. Han *et al*. [41] studied the effects of XOS on APP/PS1 mice with postoperative cognitive dysfunction. They demonstrated that treatment with XOS substantially changes the gut bacterial composition, including an increase in *Ruminococcaceae* UCG 014, *Rodentibacter*, *Bacteroides*, and *Faecalibaculum*. These alterations improve the integrity of the tight junction barrier in the intestines and hippocampus; thus, the intestinal barrier and the BBB are further protected [41].

Administering prebiotic and probiotic formulations in the diet of AD rats has favorable effects, including improved brain function caused by the reduction of gastrointestinal inflammation, OS in the hippocampus, and microglial activation [42]. Consequently, the combination of prebiotics and probiotics shows potential for enhancing the efficacy in reducing AD-related indicators.

Previous studies revealed that a high intake of prebiotics and probiotics, along with other nutrients, is associated with a slower cognitive decline and a reduced risk of AD [43]. Table 1 provides additional details regarding the diverse effects of synbiotic formulations.

## Prevention of AD by Neuroinflammation

The relationship between cytokines and AD has been established [43]. According to the amyloid cascade hypothesis, Aβ activates microglia and inflammatory cytokines [54]. In the prodromal phase of AD, soluble Aβ oligomers can decrease synaptic plasticity, impede potentiation, and stimulate microglia [55]. Neuroinflammation and pro-inflammatory cytokines enhance tau phosphorylation and decrease synaptic concentrations; consequently, cellular instability and neuronal death occur [56]. Aβ stimulates microglial receptors, which cause the release of cytokines, chemokines, and reactive oxygen species [57]. Additionally, when Aβ binds to the receptor for advanced glycation end products, inflammation occurs, and the release of pro-inflammatory cytokines such as TNFα and IL-6 is promoted [58]. Hence, the administration of anti-inflammatories can be a promising technique to prevent the incidence and progression of AD. The cytokine balance has been a focal point in understanding the etiology of AD and identifying potential new treatment targets. As illustrated in Table 1, the consumption of kefir-fermented milk considerably reduces systemic inflammation mediated by pro-inflammatory cytokines, such as IL-8, IL-12, and TNF-α [51]. TNF-α particularly participates in the inflammatory cascade and exacerbates the Aβ and tau pathology in patients with AD [59]. Short-term anti-TNF-α therapy can reduce pathology and improve cognition in these patients [60]. Similarly, the neurogenesis levels of Aβ42 in APP mice fed with only the combination of prebiotics and Vitalon Probiotics powder decrease compared with those in prebiotic-treated and control mice [49]. TNF-α levels are also reduced, but IL-1β levels remain unchanged [49].

The increased levels of the p40 subunit of IL-12 have been associated with the onset of AD [61]. Conversely, the decreased levels of IL-12 and p40 may mitigate cognitive decline and AD-related pathologies [62]. Kefir milk supplementation causes a decrease in pro-inflammatory activity and simultaneously balances pro- and anti-inflammatory cytokine levels [51]. Furthermore, IL-1β and IL-6 have been implicated in AD risk [54]. IL-1β overexpression inhibits the activity of the cholinergic system and promotes the accumulation of Aβ and NFT in AD [63]. Excessive IL-6 influences the cdk5/p53 pathway, thereby triggering tau phosphorylation [57]. Wang *et al*.[46] evaluated a combination of *Lactobacillus plantarum* 69-2 and galactooligosaccharide (GOS) by using a D-Galactose-induced aging mouse model. They showed that the levels of pro-inflammatory cytokines, such as IL-1β, IL-6, and TNF-α, substantially decrease in the combination treatment group compared with those in the groups treated with either GOS or *L. plantarum* 69-2 alone [45]. Therefore, the combination of GOS and *L. plantarum* 69-2 can activate the hepatic AMPK/SIRT1 signaling pathway by regulating the gut microbiota. The AMPK/SIRT1 signaling pathway modulates Aβ deposition and cognitive functions in AD [64]. AMPK signaling helps fight excess oxidation by mitochondrial redox processes and maintain metabolic balance [61]. The activated AMPK reduces ROS through several mechanisms because it positively affects antioxidant defense in AD [65]. In addition, regulatory information shows that the expression of silent regulator 1 (SIRT1) elicits a considerable neuroprotective effect, and SIRT1 may be a therapeutic target for AD [66]. Therefore, it not only potentially affects old mice but also extends research on mice with AD and humans.

## Prevention of AD via OS

Serum levels of OS biomarkers are increased in patients with neurodegenerative disorders; as such, they can be used to evaluate the effectiveness of novel therapeutic approaches for patients with AD [67]. ROS plays direct and indirect roles in the pathophysiology of AD. For instance, increased levels of free radicals, such as O_2_, H_2_O_2_, and NO, can damage proteins, lipids, and nucleic acids; consequently, mitochondrial degeneration and neurotoxicity occur [68]. Additionally, OS is related to Aβ-induced neurodegeneration in patients with AD [69]. BDNF serum levels are also closely correlated with AD. BDNF is an endogenous protein involved in brain function, synaptic plasticity, and structural integrity [70]. A reduced BDNF level is considered a hallmark of AD; therefore, BDNF is considered an important pathway in AD treatment [71]. In a 12-week randomized clinical trial, Haghighat et al. [52] compared the effects of consuming synbiotic supplementation with *Lactobacillus acidophilus* T16, *Bifidobacterium bifidum* BIA-6, *Bifidobacterium lactis* BIA-7, and *Bifidobacterium longum* BIA-8 with maltodextrin with the effects of taking probiotics and maltodextrin as placebo. They showed that the increase in serum BDNF levels of the group treated with synbiotic supplementation for 12 weeks was larger than that of the placebo and probiotic groups. These findings indicated that synbiotic supplementation has implications for clinical practice and future research. With the importance of BDNF in the brain and mental health, synbiotic supplementation can be used as an adjuvant therapy for patients suffering from neuropsychiatric diseases, including depression and AD [52].

Kefir treatment considerably reduces protein oxidation. Notably, kefir supplementation also decreases blood ROS availability and increases NO levels, indicating that plasma protein oxidation is reduced in patients with AD. Chunchai *et al*. [48] demonstrated that the synbiotic combination of *Lactobacillus paracasei* HIIO1 and XOS provides several beneficial effects, including the reduction of brain mitochondrial dysfunction and hippocampal OS in rats with a high-fat diet. Furthermore, this combination increases the expression of bcl-2, an anti-apoptotic protein, but it decreases the expression of bax, which is associated with apoptosis [48].

## Prevention of AD by SCFAs and Other Substances

SCFAs play a crucial role in controlling neuroinflammatory processes implicated in dementia disorders, including AD. They participate in energy metabolism and thus help compensate for any metabolic deficiencies in the brain [72]. Yilmaz *et al*. [73] found that propionate and acetic acid concentrations in patients with AD were respectively 1.35 and 1.25 times higher than those in the control group. The levels of seven different SCFAs remarkably decrease in fecal samples of patients with AD [74]. SCFAs may influence not only the development and function of microglia in the CNS but also the self-assembly of Aβ peptides in patients with AD [75]; as such, they are promising candidates for effectively suppressing AD. Fernando *et al*. [76] investigated the effects of oral sodium butyrate treatment on the brain of 5xFAD mice. They showed that Aβ levels decrease in a dose-dependent manner during early disease progression. Sodium propionate also prevents Aβ-induced neurotoxicity by decreasing the production of stimulated COX-2 [77].

Westfall *et al*. [78] used a Triphala formulation rich in polyphenols that act as a prebiotic agent and combined it with probiotic bacteria (*L. plantarum* NCIMB 8826, *L. fermentum* NCIMB 5221, and *B. longum* spp. *infantis* NCIMB702255); they found that this combination modulates the PPAR pathway and free fatty acids. In another study, this synbiotic combination may positively affect the incidence and progression of AD in a transgenic humanized *Drosophila melanogaster* model [47]. *L. fermentum* promotes the conversion of fatty acids to caffeic acid and vanillic acid, which strengthen the anti-inflammatory characteristics of the mixture and provide neuroprotective effects against fibril formation. Furthermore, the prebiotic TFLA contains high gallic acid levels, which slow down neuronal degeneration and maintain a healthy gut flora. *L. plantarum*-GOS produces antibacterial compounds and SCFAs suppressing harmful bacteria, enhances intestinal mucosal thickness, and increases mucin levels; thus, it helps reduce the likelihood of chronic neuro-inflammation, which is one of the major risk factors for AD progression [46]. The activation of the AMPK/SIRT1 response pathway via the gut–liver axis also induces OS [48].

Depression is considered an early symptom and risk factor for AD diagnosis. Individuals who have experienced depressive episodes in the past more likely develop AD later in life compared with those without a history of depression [79]. This observation highlights the importance of mental health in the context of neurological disorders. Furthermore, changes in the gut microbiota have been associated with variations in the progression of neurodegenerative diseases, including AD [80]. Tamtaji *et al*. [50] administered a 12-week treatment using synbiotic and probiotic therapy (selenium with probiotics) to patients with AD; they showed that metabolic abnormalities, inflammation, and OS in these patients improved compared with those in the placebo group. Specifically, the 12-week selenium supplementation reduced the serum high-sensitivity C-reactive protein, homeostasis model of assessment insulin resistance, and LDL-cholesterol and total-/HDL-cholesterol ratio; conversely, it significantly increased the total GSH levels and the quantitative insulin sensitivity check index. In addition, the mini-mental state examination scores increased in patients treated with the selenium supplementation with probiotics compared with those in patients treated with probiotics and selenium alone [50].

## Future Concerns about Synbiotics for the Prevention of AD

Synbiotic medication shows therapeutic potential for AD; however, several limitations should be addressed before it can be recommended in medical practice. First, consuming probiotics has raised concerns on its potential adverse effects, including sepsis, hypersensitivity reactions, and gene transfer leading to pathogenic antibiotic resistance [81]. Another study specifically warns against the use of probiotics in patients with AD receiving immunosuppressive therapies such as chemotherapy because of cases of fungal blood infections and sepsis in recipients of *S. boulardii* [82]. Certain probiotic strains, including those that can cause infections, may carry genes for antibiotic resistance [82].

Second, the effects of probiotics exhibit individual variability [83]. Because of this variability, determining which specific probiotic within a multi-probiotic treatment is responsible for observed outcomes becomes challenging. Some studies have suggested that using probiotics after antibiotic treatment can increase the risk of contracting infectious diseases by causing persistent dysbiosis [84]. Probiotics can metabolize tryptophan, which substantially raises the risk of serotonin syndrome in combination with potent selective serotonin reuptake inhibitors [41]. The effects of serotonin syndrome can range from mild symptoms, such as shivering and diarrhea, to severe manifestations, including muscle rigidity, fever, seizures, and even death [85]. Therefore, a limited number of studies have investigated the application of probiotics and synbiotics as a medical intervention for AD. Extreme caution should be exercised when considering their use in humans, particularly in the elderly and individuals with multiple comorbidities.

Lastly, the potential negative effects of prebiotics on AD have not been extensively studied. However, certain prebiotics may contribute to neuroinflammation. For example, lactulose, a prebiotic, is associated with reduced short-term memory and impaired learning capacity in mice with AD [86]. To address these concerns and mitigate potential risks, researchers should conduct further long-term studies on the mechanisms, benefits, and drawbacks of synbiotics. Obtaining additional evidence supporting their safety and efficacy will enhance their reliability and maximize their potential for pharmaceutical and medical applications to prevent and manage AD.

## Conclusion

Synbiotics have shown promising results in improving microbiome stability and surpassing the individual use of probiotics or prebiotics alone. They positively affect brain health, particularly the prevention of neurodegenerative diseases such as AD. They may mitigate or even reverse the primary symptoms of AD through various mechanistic pathways, such as reducing chronic neuroinflammation, modulating peripheral factors, influencing the production and translation of neurotoxic metabolites, regulating gut microbiota, and reducing OS. However, further studies should be performed to fully understand the etiology of AD and reveal the mechanisms by which synbiotics exert their effects.

The encouraging outcomes described in this review support the potential use of synbiotics as an effective medical therapy for AD. Nonetheless, further research is required to elucidate their precise mode of action and establish stronger connections between microbial and AD research. Continued investigation will help clarify their therapeutic mechanisms and optimize their application in AD prevention and treatment.

## Figures and Tables

**Table 1:** Effects of synbiotics on AD models.

Synbiotics	Subject/Model	Dose and duration	Effects	References
**In vitro experiments**				
*Lactobacillus plantarum*, *Bifidobacterium infantis*, and *L. salivarius* with grapeseed polyphenolic extract	In vitro model of the human gastrointestinal tract	*N/A*	■ Inhibited the aggregation of amyloid plaques and tau fibrils ■ Exhibited anti-inflammatory activities	Pasinetti [44]
**In vivo experiments on mice**				
*L. plantarum* 69-2 with GOS	Male BALB/c mice aged 8 weeks were injected with 50 mg/ml Dgalactose per day for 6 weeks	■ 500 mg/kg GOS every day ■ 5×10^9^ CFU kg/l *L. plantarum* 69-2 per day ■ Combination of 500 mg GOS and 5 × 10^9^ CFU *L. plantarum* 69-2 daily	■ Increased the total antioxidant capacity (T-AOC) and glutathione (GSH) activities, leading to enhanced antioxidant capacity in the liver ■ Decreased the amount of *Proteobacteria* in the stomach and considerably increased the quantity of *Verrucomicrobia* and the levels of SCFA (acetate, propionate, and butyrate) ■ Repressed the gene expression of AMP-activated protein kinases (AMPK1 and AMPK2) and sirtuin 1 (SIRT1) and significantly increased the levels of aging-related genes, such as Bax, P53, and nuclear factor kappa-B (NF-κB), which might be attributed to the promotion of the AMPK/SIRT1 pathway, potentially regulating the expression of aging-related genes	Wang *et al*. [45]
*L. plantarum* NCIMB 8826, *Lactobacillus fermentum* NCIMB 5221, and *Bifidobacterium longum* spp. *infantis* with *Emblica officinalis*, *Terminalia bellirica*, and *Terminalia chebula* powder	*Drosophila melanogaster* with transgenic BACE1-APP gene	■ 3.0 × 10^9^ CFU/ml of each type of bacteria mixed with 0.5% of TFLA powder for 30 days	■ Slowed down acetylcholinesterase growth and improved survival ■ Inhibited the development of the pathogens *S. aureus* and *E. coli* ■ Increased the amount of total triglycerides because of the increased gene expression of acetyl CoA carboxylase after 30 days ■ Decreased the expression of lipid peroxidation	Westfall *et al*. [46]
*Lactobacillus paracasei* *HII01* with XOS	Male Wistar rats fed with a high-fat diet or normal diet	■ 2 ml of the combination of 10% XOS and 10^8^ CFU *L. paracasei* HII01 for 12 weeks	■ Reduced the mRNA expression levels of the proinflammatory cytokines interleukin (IL)-1 and IL-6 ■ Prevented hippocampal dysplasia via LTP development and enhanced the dendritic spine density ■ Improved brain mitochondrial dysfunction, reduced hippocampus oxidative stress, and elicited anti-apoptotic effects	Chunchai *et al*. [47]
*Lactobacillus rhamnosus* *UBLR-58* with curcumin	Mice with scopolamineinduced memory impairment	■ 1 ml/kg/day for 10 days	■ Increased antioxidant enzyme levels ■Minimized neuronal damage ■ Improved memory and cognitive functions in the behavioral study	Patel *et al*. [48]
Prebiotics mixed with Vitalon Probiotics powder (v/v: 7/1)	APP transgenic mice	■ 4.1 g/kg/day for 2months	■ Decreased the accumulation level of Aβ42 ■ Did not change Aβ40 ■ Reduced the TNF-α gene expression but did not affect IL-1β	Deng *et al*. [49]
**In vivo experiments on humans**				
*Lactobacillus acidophilus*, *Bifidobacterium bifidum*, and *B. longum* with selenium	Patients with AD	■ 200 μg/day for 12 weeks	■ Improved cognitive function ■ Influenced the MMSE score, hs-CRP, TAC, GSH, insulin metabolism markers, triglycerides, VLDL-, LDL-, and total-/HDL-cholesterol ■ Improved the gene expression of TNF-a, PPAR-g, and LDLR	Tamtaji *et al*. [50]
Milk supplemented with kefir grains	Human	■ 2 ml per kilogram of body weight every day for 90 days	■ Favored cognitive improvement in the memory, language, construction ability, and attentional function assessments of patients after 90 days ■ Decreased the levels of O_2_-, H_2_O_2_, and ONOO-/OH- levels and increased the levels of NO and the oxidation–reduction of proteins	Ton *et al*. [51]
*Lactobacillus acidophilus* T16, *B. bifidum* BIA-6, *Bifidobacterium lactis* BIA-7, and *B. longum* BIA with maltodextrin	Patients with high depression	■ 5 g of probiotics, 5 g of synbiotics, and 20 g of prebiotics in for weeks	■ Significantly increased the brain-derived neurotrophic factor in the synbiotic group compared with that in the probiotic and maltodextrin-only groups	Haghighat *et al*. [52]
